# Sealing Ability of Mineral Trioxide Aggregate and Calcium-Enriched Mixture Cement as Apical Barriers with Different Obturation Techniques 

**Published:** 2014-10-07

**Authors:** Mahdi Tabrizizade, Yasin Asadi, Aidin Sooratgar, Saeed Moradi, Hossein Sooratgar, Fatemeh Ayatollahi

**Affiliations:** a *Department of Endodontics, Dental School, Shahid Sadoughi University of Medical Sciences, Yazd, Iran;*; b *Department of Periodontics, Dental School, Shahid Sadoughi University of Medical Sciences, Yazd, Iran;*; c *Department of Endodontics, Dental School, Mashhad University of Medical Sciences, Mashhad, Iran;*; d *Dental School, Mashhad University of Medical Sciences, Mashhad, Iran*

**Keywords:** Apexification, Apical Plug, Apical Seal, Calcium-Enriched Mixture, Mineral Trioxide Aggregate, Microleakage, Root Canal Obturation, Root Canal Sealing Material

## Abstract

**Introduction:** Endodontic treatment in pulpless immature teeth is challenging due to the lack of an apical stop. Insertion of an apical plug is an alternative to conventional long-term apexification with calcium hydroxide. The aim of this study was to compare the apical microleakage of mineral trioxide aggregate (MTA) and calcium-enriched mixture (CEM) cement as apical plugs with three different obturation techniques. **Methods and Materials:** This experimental study was conducted on 130 single rooted human teeth with one canal. Samples were randomly divided into 2 experimental groups (*n*=60) and two negative and positive control groups containing 5 samples each. After cleaning and shaping, an open apex configuration was prepared in all samples. MTA or CEM cement apical plugs with 5 mm thicknesses were placed. Then, each group was divided to 4 subgroups and the remaining space of root canals were filled with either lateral compaction or thermoplasticized injectable gutta-percha or was obturated by filling the entire canal with apical plug material. In one remaining subgroup the canal space was left unfilled. Microleakage was measured by the fluid filtration method and results were analyzed by means of the two-way ANOVA test. **Results: **There were no significant differences between microleakage of MTA and CEM cement apical plugs (*P*=0.92). The difference between three obturation methods was not significant, either (*P*=0.39). **Conclusion: **MTA and CEM cement have similar sealing ability as apical plugs and no significant difference was found in microleakage of the three groups.

## Introduction

Endodontic treatment of an open-apex tooth with a necrotic pulp has always been challenging. For many years, calcium hydroxide (CH) has been advocated for apexification and induction of an apical hard tissue matrix which can limit the obturating material within the canal space [1]. The main drawback of this procedure is the need for multiple appointments and this long-term treatment procedure makes the tooth susceptible to fracture [2, 3]. Therefore, one-step apexification or apical plug technique was suggested. There are many reports that disclose successful treatment of the open-apex teeth by means of mineral trioxide aggregate (MTA) as an apical plug. Several review articles have also described clinical procedures with MTA as an apical plug [4]. MTA is a biocompatible material [5, 6] with few drawbacks like long setting time, tooth discoloration potential [4], and difficult manipulation [7].

Another biomaterial for this purpose is calcium-enriched mixture (CEM) cement which consists of different calcium compounds [8]. CEM cement has good sealing ability as retrograde filling material [9] and has shown satisfactory pulpal response as a direct pulp capping agent [10].

An important factor for successful endodontic treatment in open-apex teeth is the sealing ability of apical plug. Few studies have compared the sealing ability of MTA and CEM cement as root-end filling or furcal perforation repair materials; CEM cement has shown a similar, if not superior, sealing ability compared to MTA [11-13].

Comparison of the subcutaneous tissue responses to CEM cement, with white and gray ProRoot MTA (Dentsply, Tulsa Dental, Tulsa, OK, USA) showed that CEM does not induce tissue necrosis and histological observation illustrated that ProRoot MTA and CEM cement were well tolerated by the subcutaneous tissues [14].

Antimicrobial assessments have revealed that CEM cement is a potent antibacterial agent like CH and they are both significantly superior to MTA in this regard [15]. In physical evaluations, CEM exhibited shorter setting time, more flowability and considerably less film thickness than MTA [16]. Moreover CEM cement has good handling properties and is not sticky, so it does not adhere to the applicator and is condensed easily [13]. Considering the mentioned properties of CEM cement, it can potentially be the material of choice in endodontic treatment of open-apex teeth.

It is clear that successful endodontic treatment requires an adequate seal in entire root canal length which includes coronal, lateral and apical seal. So the obturation of remaining root canal space above the apical plug is important. Several studies compared different obturation methods in teeth with mature apices [17, 18], but few studies have compared them in open-apex teeth after placement of the apical plug [19], so the present study compared the apical microleakage of MTA and CEM cement apical plugs with three different methods of obturation in open-apex teeth.

## Methods and Materials

This experimental study was conducted on a total of 130 single rooted human teeth with single canals. Teeth with caries, resorption, severe root curvature and infraction were excluded.

Attached calculus and soft tissues were removed with a periodontal curette (Juya, Keshmir, Pakistan). For disinfection, the teeth were stored in 5.25% sodium hypochlorite (NaOCl) for 1 h and then placed in normal saline. The samples were decoronated with high speed diamond bur (010, Tizkavan, Tehran, Iran) so that the roots had approximately 12 mm length.

Root canal lengths were navigated by inserting a #15 K-ﬁle (Dentsply Maillefer, Ballaigues, Switzerland) into the canals, until the instrument tips were visible at the apical foramen. Working length (WL) was determined 1 mm shorter than this length. Cleaning and shaping was carried out with stainless steel K-ﬁles up to #40 as the master apical ﬁle and then canals were ﬂared up to #80 with step back technique. After using each instrument, the canals were irrigated with 1.0 mL of 2.5% NaOCl. To simulate an open-apex shape, the apical foramina were enlarged with Peeso drills (Dentsply, Maillefer, Tulsa, Ok, USA) sizes 1 to 4 in an orthograde manner, which resulted in apical foramen with 1.3 mm diameter. To remove the smear layer, canals were filled with 3.0 mL of 17% Ethylenediaminetetraacetic acid (EDTA) (Ariadent, Tehran, Iran) and 1 mL of 2.5% NaOCl for 3 min, each. Final irrigation was done with 5 mL of normal saline.

These steps were similar for all samples. Subsequently samples were randomly divided in two experimental groups, *i.e.* MTA and CEM (*n*=60), and two positive and negative control groups with five samples each. The groups were further divided into 4 subgroups of 15 teeth each. Next steps in different groups were carried out as follows: Roots were fixed in flower mounting sponges and moist cotton was placed opposite the apices to simulate periapical soft tissues. Canals were dried with #80 paper points (Ariadent, Tehran, Iran). Angelus MTA (Angelus, Londrina, PR, Brazil) was prepared according to the manufacturer’s guideline and was carried into the canals with MTA carrier (Dentsply Maillefer, Ballaigues, Switzerland) and condensed up to the apical end with #3 and 4 hand pluggers (Dentsply Maillefer, Ballaigues, Switzerland) with a rubber stop positioned 5 mm shorter than the WL. The excess material was removed and 5 mm thick apical plugs were condensed. Then a moistened paper point was placed in the canals and then the density and thickness of apical plugs were confirmed by taking radiographs. Access cavities were restored with temporary restoration material (Coltosol; Ariadent, Tehran, Iran) and samples were stored in 37^º^C and 100% humidity for 24 h.

In subgroup1 after removing the temporary restoration and checking proper setting of MTA, the remaining canal space above the apical plug was filled with lateral condensation of gutta-percha (Diadent, Chongju, Korea) and AH-26 sealer (Dentsply DeTrey, Konstanz, Germany). In subgroup 2 the remaining canal space was filled with injectable thermoplasticized gutta-percha and AH-26 sealer using the injection device (Obtura II system, Obtura/Spartan, Fenton, Missouri, USA). In Subgroup 3 the entire canal space was filled with MTA. In subgroup 4 the canal space was left unfilled. Similar steps were carried out for 4 subgroups of CEM group in which CEM cement (BioniqueDent, Tehran, Iran) that was mixed and used according to the user’s manual.

In negative control samples, canals were filled with gutta-percha and the entire root surface including the apical foramen was covered by 2 coats of nail polish and sticky wax (Kerr, Berlin, Germany). Positive control samples were papered and canals were left totally unfilled. In experimental and positive control samples, external root surfaces except for the apical 2 mm, was covered by 2 coats of nail polish. All samples were stored in 37^º^C and 100% humidity for 48 h.

To eliminate inter-examiner variables all these steps were carried out by one person. Leakage was evaluated using the ﬂuid ﬁltration method as described by Moradi *et al.* [20]. The root surfaces were covered with two layers of parafilm except for the apical 2 mm. The roots were then connected to a plastic tube with cyanoacrylate glue (Santen Pharmaceutical Co., Osaka, Japan) at the apical side and were additionally sealed with parafilm. A plastic three valve adaptor was connected to the other side of the plastic tube. A standard glass capillary tube was connected to the three valves. All pipettes, syringes and the plastic tubes at apical sides of the specimens were filled with distilled water. Using a syringe, water was sucked back and air bubbles were created. A pressure of 0.5 atm was applied at the end of the capillary tube to force the water through the voids along the filling, thus displacing the air bubble in the capillary tube. All junctions were sealed by cyanoacrylate and parafilm.

**Table1:** Main (SD) of microleakage (µL/min/cm H_2_O) in different groups (AP=apical plug, GP=gutta-percha, TO=thermoplasticized obturation)

**Variable**		**Mean (SD)**	***P*** **-value**
**Material**	**CEM cement**	68* (28)	0.92
	**MTA**	67 (39)	
**Subgrou** **ps**	**AP+GP**	55 (28)	0.005
	**AP+TO**	67 (14)	
	**Obturation with AP material**	56 (29)	
	**Unfilled**	96 (37)	

The volume of the fluid transport was measured by observing the movement of the air bubble. The observation was done by a digital camera (Olympus, C765, Tokyo, Japan) stabilized in a distinct distance from the micropipette.

The first observation was done 30 sec after pressure for localization of the bubble and then digital photographs were taken in 2-min intervals at 2, 4, 6, and 8 min. Finally, a software was used for measuring the bubble movement and the data were calculated in µL/min/cm H_2_O. The Kolmogorov-Smirnov test was used in order to determine the normality of dispersal distribution of parameters; thereafter, results were analyzed by the mixed ANOVA. The level of significance was set at 0.05.

## Results

The positive control group showed the maximum amount of leakage, while the negative control group did not show any leakage. The samples in experimental groups demonstrated different amounts of apical leakage. Table 1 demonstrates the main and interaction effect of variables on microleakage.

Based on the statistical analyzes of data, there was no significant difference between the test materials (*P*=0.92). There was a significant difference between microleakage of subgroups 1 to 3 and 4 (*P*=0.005) but the difference between the microleakage of three mentioned methods in subgroups 1, 2 and 3 was not statistically significant (*P*=0.39).

**Figure 1:**
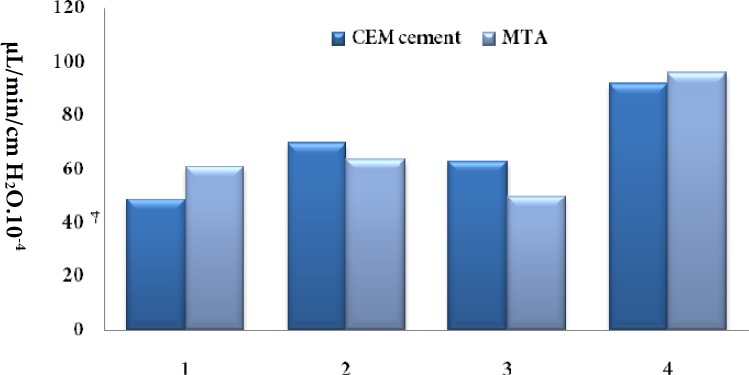
Microleakage in different subgroups of MTA and CEM cement

In MTA group, subgroup 3 showed the lowest microleakage (50×10^-4^ µL/min/cm H_2_O) while the 4^th^ subgroup showed the greatest amount of microleakage (96×10^-4^ µL/min/cm H_2_O). In CEM group the lowest and highest amount of microleakage was observed in subgroups 1 (49×10^-4^ µL/min/cm H_2_O) and 4 (92×10^-4^ µL/min/cm H_2_O), respectively (Figure 1).

## Discussion

This study compared the microleakage of MTA and CEM cement as apical barriers with three different obturation techniques. Various methods have been suggested to evaluate the sealing ability of apical plugs, such as dye leakage, bacterial leakage, glucose penetration, radioisotope labeling, electrochemical method and ﬂuid ﬁltration [21].

Dye penetration method is widely used but it has some potential problems. Particle molecular size, pH and chemical reactivity of the dye can affect its degree of penetration [21]. In addition, it was shown that alkaline materials such as MTA and CEM cement can discolorate the methylene blue dye, which may lead to unreliable findings [22]. The use of bacteria to assess leakage seems more clinically and biologically relevant than the dye leakage methods [23]. Bacterial leakage tests are qualitative in nature because one passing bacteria through the root canal can multiply and cause positive culture and turbidity [21]. Moreover if canal filling materials have antimicrobial properties, like MTA and CEM cement [15, 24], it is irrational to employ this method [21].

One of the potential problems of tracers in leakage studies, is the chemical reactivity of tracer with filling materials and probability of obtaining unreliable results [21]. Glucose penetration method was introduced for endodontic leakage studies [25]. This method evaluates the quantitative cumulative longitudinal microleakage based on the filtration rate of the glucose along the filled root canal. A recent study showed that glucose in alkaline solutions is slowly oxidized; forming gluconic acid that finally converts to gluconate, and glucose detection kits cannot detect gluconate. Therefore, the sealing ability of alkaline materials like MTA should not be evaluated with the glucose leakage model [26].

In the present study, microleakage was evaluated by fluid filtration technique. In this method samples are not destroyed, so the longitudinal sealing ability can be assessed. Since very small volume can be recorded in this method, the results are accurate and no tracer is needed that eliminates the potential problems of molecular size, chemical reactivity or pH [27].

In the present study there was no significant difference between the mean microleakage in MTA and CEM groups. Similar results were reported by Asgary *et al.* [9] who compared the sealing ability of 3 types of MTA and CEM cement as root-end filling materials by dye penetration method. Yavari *et al.* [28] also compared the sealing ability of four dental materials as intra-orifice barriers by polymicrobial leakage method. They found that MTA and CEM cement have superior sealing ability than composite resin and amalgam; however they had nosignificant difference with each other. Kazem *et al.* [29] showed that CEM cement, Root MTA and White ProRoot MTA have similar microleakage as root-end filling materials using dye and bacterial penetration method.

However, some studies have reported superior sealing ability for CEM cement [12, 13, 30]. Perhaps, the differences in sample size, tooth type, timing and methods of the microleakage assessment in these studies can explain the different results.

In one-step apexification, after placement of the apical plug, there are different methods for filling the remaining canal space. Lateral compaction, as the most common used obturation method, can be the first choice. However, with this method a dense and homogenous obturation in a wide canal space of an open-apex tooth may be challenging. In addition, forces applied by the spreader may results in root fracture of immature teeth [31, 32]. In addition, using more sealer to fill the voids increases the risk of subsequent sealer dissolution [33].

Thermoplasticized gutta-percha is another option for filling the remaining canal space that results in more homogenous filling of wide canal space without extrusion of the filling materials in presence of apical plug. However, it requires specific equipments that may not be available in all dental centers. Obturation of the entire root canal with apical plug material is another option for obturation of the open-apex teeth [34].

MTA as a reliable bioactive material has extended applications in endodontics, which include the obturation of the root canal space. Clinical case reports have documented clinical outcomes after application of MTA as filling material in different circumstances and demonstrated its effectiveness in resolving apical periodontitis [35, 36].

In the present study, three methods for obturation of open-apex teeth, lateral compaction or thermoplasticized gutta-percha after placement of MTA or CEM cement apical plugs and filling the entire canal with apical plug materials showed no significant difference in microleakage.

Vizgirda *et al.* [33] compared three obturation methods and found no significant differences in leakage between the lateral compaction and the thermoplasticized gutta-percha method. An evaluation of leakage associated with three root-filling techniques in large and extremely large root canals showed no significant differences between cold lateral condensation and thermoplastic compaction [37].

Vizgirda *et al.* [33] also compared the quality of apical sealing by lateral and thermoplasticized obturation with gutta-percha to MTA obturation. The results suggested that gutta-percha obturation may provide an apical seal that is superior to MTA. In the present study microleakage in canals completely filled with MTA or CEM did not differ significantly with those treated with apical plug and gutta-percha. Martin *et al.* [19] evaluated the microleakage in open-apex teeth that were filled with MTA or treated by MTA apical plug and thermoplasticized gutta-percha. They found that roots with MTA fillings exhibited a better seal than MTA apical plugs at 48 h; however seal of two groups was not significantly different after 4 weeks.

In this study samples of subgroup 4 which had only 5 mm-thick apical plugs, showed the greatest amount of microleakage that seems rational considering the lower thickness of canal filling, as Adel *et al.* [12] found that increasing the apical plug thickness increases the sealing ability of apical barriers.

Within the limitations of the present experimental study, it can be concluded that one visit obturation of open-apex teeth with MTA or CEM cement may be a suitable alternative for apical plug placement and gutta-percha obturation. It doesn’t require special equipments and doesn’t have potential problems associated with lateral forces in lateral condensation method. However difficulty of removing the filling material in cases of retreatment or post placement and also discoloration potential [12, 24] in anterior teeth should not be overlooked. Future clinical investigations would provide further support to these findings.

## Conclusion

According to results of this study, MTA and CEM cement have similar sealing ability as apical plugs with various obturation techniques.
